# Effectiveness of interventions for social isolation, loneliness, and social participation in older adults with hearing loss: results from a systematic review

**DOI:** 10.1186/s13643-026-03107-y

**Published:** 2026-02-16

**Authors:** Julie Beadle, Esther Yuwono, Mélanie Levasseur, Andrew Wister

**Affiliations:** 1 Gerontology Research Center, Simon Fraser University, 2800-515 West Hastings St., Vancouver, Canada; 2 Department of Gerontology, Simon Fraser University, 2800-515 West Hastings St., Vancouver, Canada; 3 School of Rehabilitation, Faculty of Medicine and Health Sciences, Université de Sherbrooke, Sherbrooke, Canada; 4 Research Centre On Aging, Eastern Townships Integrated University Health and Social Services Centre, Sherbrooke University Hospital Centre, Sherbrooke, Canada

**Keywords:** Intervention, Hearing loss, Older adults, Social isolation, Loneliness

## Abstract

**Background:**

Hearing loss is increasingly prevalent among older adults and significantly impacts their ability and motivation to communicate. This often results in reduced social participation, increased feelings of loneliness, and a higher risk of social isolation. This review aims to evaluate the effectiveness of interventions designed to improve social participation, reduce loneliness, and mitigate social isolation in older adults with hearing loss.

**Methods:**

This review followed PRISMA guidelines. Keyword and subject heading searches were conducted within MEDLINE, EMBASE, PsycINFO, AgeLine, CINAHL, and ProQuest Sociology. Articles were selected based on pre-defined inclusion and exclusion criteria. Quality assessment was conducted with the Cochrane Risk of Bias and the Risk of Bias in Non-randomized Studies of Interventions tools.

**Results:**

The search identified 746 records, of which 11 met inclusion criteria. Two studies employed pilot randomized controlled trial designs, while the remaining studies used prospective pre–post observational designs. Interventions included hearing aids, cochlear implants, assistive listening technologies, and Group Auditory Rehabilitation (GAR). Across study designs, interventions targeting hearing loss were consistently showed improvements in loneliness and social participation, with the strongest and most consistent evidence observed for GAR combined with hearing device uptake. In contrast, social isolation was less frequently measured, limiting conclusions for this outcome relative to loneliness and social participation.

**Conclusions:**

The best available evidence across diverse study designs suggests that hearing interventions, including hearing devices and Group Auditory Rehabilitation, can support improvements in loneliness and social participation in older adults with hearing loss. Although fewer studies have directly examined social isolation, available findings indicate potential benefit. Future research should further evaluate social isolation outcomes, long-term sustainability, and mechanisms of change.

**Systematic review registration:**

PROSPERO database (reference number CRD42024529695).

Social isolation and loneliness are common experiences worldwide, particularly for older adults [1]. The National Institute on Aging reports that for Canadians aged 50 and older, up to 58% experience some degree of loneliness, and that 41% are at risk for social isolation [2]. Although social isolation and loneliness have been measured using many different instruments, sometimes together, they are different concepts. Social isolation has been defined as an “*objective measurable state capturing the level and frequency of one’s social interactions*” [3] and “*having few social relationships or infrequent social contact with others*” [4], whereas loneliness as the subjective “*feeling that accompanies the perception that one’s social needs are not being met by the quantity or especially the quality of one’s social relationships*” [5]. Social isolation and loneliness can be limited by increasing social participation, which represents the “*person’s involvement in activities that provide interactions with others”* [6]* “in community life and in important shared spaces, evolving according to available time and resources, and based on the societal context and what individuals want and is meaningful to them*” [7]. According to these operational definitions, social isolation, loneliness and restriction in social participation often co-occur; however, it is possible to be socially isolated or not participate but not feel lonely, and to be lonely but not socially isolated nor restricted in social participation [7–9].

One important factor influencing social participation, isolation or loneliness for many older adults is hearing loss. Current estimates suggest that approximately half of adults aged 65 and older have some level of hearing loss, with its prevalence and severity increasing with age [10, 11]. Mild hearing loss also impacts older adults’ ability to remain socially engaged, particularly in situations with background noise (*e.g.*, a busy *café* or recreation centre). Untreated hearing loss may lead to social withdrawal, social isolation, and loneliness [12, 13].

Focusing on the older adult population, several studies have identified an association between hearing loss and social isolation [14–17] and hearing loss and loneliness [18–21]. A recent scoping review on social participation found that older adults with hearing loss face significant challenges in maintaining relationships and participating in social activities. While social support and active coping strategies were identified as key facilitators of social engagement, the review highlighted that barriers such as increased hearing loss, communication difficulties, and comorbid health conditions often impede their ability to stay connected [22]. As proposed by Motala et al. [23], progressive changes in social behaviours connected to hearing loss likely influence the trajectory of social participation restriction and social isolation over time, such as withdrawing from conversations, fatigue, ‘zoning out’, or increased avoidance of social situations due to difficulties understanding others.

Loneliness and social isolation have also been associated with higher risk of disease and mental illness, including a 29% increased risk of heart disease, 32% stroke [24], 25% cancer mortality, 59% functional decline [25], 50% dementia [2, 24, 26], anxiety, depression [27], and susceptibility to viruses and respiratory illnesses [28]. Social isolation and loneliness also increase the risk for premature death by 29% and 26%, respectively [29]. Therefore, it is critical to identify effective interventions to minimize social isolation, loneliness, and restriction in social participation in older adults, particularly for those with health conditions that interact with communication, such as hearing loss. This review thus aimed to assess the effectiveness of interventions for social isolation, loneliness, and social participation in older adults with hearing loss. As two previous reviews on this topic have only focused on hearing-specific interventions [12, 30], the current review aimed to examine whether any other types of interventions have been successfully implemented for older adults with hearing loss, and if significant updates to the field, including RCTs, are available within this area of research.

## Methods

This systematic review followed the guidelines from the Preferred Reporting Items for Systematic Reviews and Meta-Analyses (PRISMA) 2020 Statement [31]. The review protocol was registered in the International Prospective Register of Systematic Reviews (PROSPERO) database (reference number: CRD42024529695).

### Inclusion and exclusion criteria

Following the PICOS framework, the research question for the review was identified as: How effective are interventions for social isolation, loneliness, and social participation in older adults with hearing loss? Adults aged 60 and older were included in the participant sample (or the results for this age group were reported separately such that this data could be extracted). Participants had a confirmed diagnosis of hearing loss. Studies reporting data from only participants less than 60 years of age, or from participants without a diagnosed hearing loss, were excluded. This review included all types of interventions, encompassing both hearing-focused approaches (e.g., hearing aids, cochlear implants, auditory rehabilitation) and interventions not specifically aimed at addressing hearing impairment (e.g., exercise). Studies quantitatively measuring social isolation, loneliness, or social participation as primary or secondary outcome variables, using standardized or non-standardized assessments, were included. Studies not measuring social isolation, loneliness, or social participation were excluded (*e.g.*, assessing Quality of Life). This review included experimental and prospective observational study designs, with or without comparison or control groups. RCTs, non-RCTs, and single group pretest-postest intervention designs were included. Pretest–posttest studies relying on retrospective data collection were excluded to reduce the risk of recall bias.

### Information sources and search strategy

In consultation with a reference librarian, we identified six academic databases for the search: MEDLINE, EMBASE, PsycINFO, AgeLine, CINAHL, and ProQuest Sociology Collection. For all databases, searches were limited to peer reviewed full texts available in English and dated 2000—2024. The ProQuest Sociology search was run on January 30, 2025; all other searches were run on January 25, 2025. The keywords in Table 1 were used for PsycINFO, AgeLine, CINAHL, and ProQuest Sociology searches. As advised by the reference librarian, both Keywords and Medical Subject Headings (MeSH) were used for MEDLINE and EMBASE searches. The search protocol for each database is available in Appendix A.

**Table 1:** Example keyword search strategy

Search	Keywords
1	seniors OR elderly OR "older persons" OR "older adults" OR gerontolog* OR geriatr* OR aging
2	"hearing loss" OR deaf* OR "hearing impair*" OR "hard of hearing" OR "hearing disorders"
3	intervention OR "program evaluation" OR trial OR "hearing rehabilitation" OR "hearing aid" OR "cochlear implant" OR "assistive listen*"
4	loneliness OR "social isolation" OR "social participation" OR "social engagement" OR "social activit*" OR "social network*" OR "social interaction*" OR "social disengagement" OR "social alienation"
5	S1 AND S2 AND S3 AND S4

All articles resulting from our search were uploaded to the *Covidence* data management platform to eliminate duplicates and for subsequent screening. Following de-duplication, two reviewers (JB and EY) independently screened all remaining titles and abstracts for eligibility by applying study inclusion/exclusion criteria (see Table 2). Any conflicts were resolved by a third research team member (AW). Articles identified for full review were gathered through institutional journal subscriptions or inter-library loan. Two reviewers independently reviewed the full-texts of records that passed screening, applying study inclusion/exclusion criteria, with any full-text screening conflicts resolved by a third research team member. Hand searching was completed with web of science, google scholar, and reference lists.

**Table 2:** Inclusion and exclusion criteria

Inclusion criteria	Exclusion criteria
1) Published in English, peer reviewed and from 2000—2024 2) Adults aged 60 and older with a diagnosis of hearing loss are included in the participant sample (or the results for this age group were reported separately such that this data could be extracted) 3) An intervention is included 4) Social participation/isolation or loneliness, is measured in some manner, pre and post intervention	1) The full text is not available 2) The record is a review paper 3) Formal statistical analysis was not completed on the intervention effect (intervention effect not reported)

### Data extraction, evaluation, and synthesis

A data extraction template was developed, following the *Cochrane Handbook for Systematic Reviews of Interventions *[32] and the *Covidence Guide: Extraction for Intervention Systematic Reviews* [33]. All authors reviewed the template. The extracted information included general information (author, full reference, country), population characteristics (sample size, age, sex, hearing loss severity, comorbidities), method (study aims and design, time points, intervention type, missing data, and attrition), outcome measures, results, funding, and possible conflicts of interest. Two reviewers independently completed data extraction for each record. Reviewers met weekly to discuss each extraction and achieve consensus. To determine the risk of bias for each study, RCTs were assessed using the Cochrane Risk of Bias tool [34], and non-randomized studies were assessed using the Risk of Bias in Non-randomized Studies of Interventions (ROBINS-I) tool [35].

## Results

**Fig. 1:**
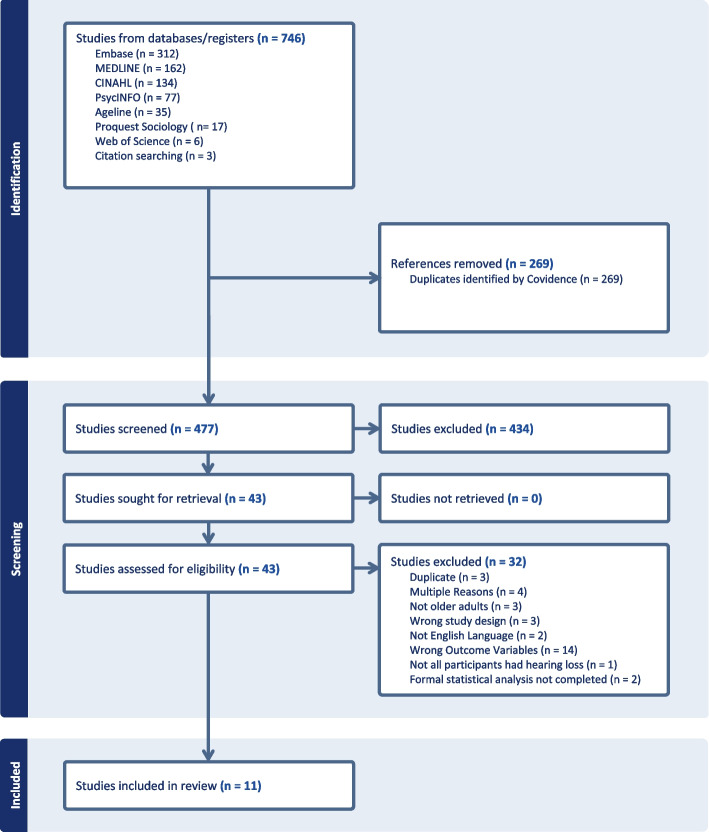
PRISMA

Figure 1 (PRISMA) summarizes the results at each stage of the review process and reasons for study exclusion. Out of the 746 records imported to Covidence, 269 were identified as duplicates. The abstracts and titles of the remaining 477 studies were screened. Forty-three full texts were assessed for eligibility, with 32 studies excluded at the full text screening stage. The 11 studies included in this review are summarized in Tables 3 and 4. Due to the variety of outcome measures, different covariates, and limited RCTs, it was not possible to conduct a meta-analysis with the selected studies. A narrative synthesis is therefore included below.

**Table 3:** Randomized controlled trial (RCT) studies

Study	Group	Timepoint	Sample			Social outcome measure(s)	Intervention	Results summary
			Size (Males)	Age	Hearing loss			
Jones et al. 2019 [41]	Intervention	Baseline	*n* = 35 (21)	M (SD) 74.3 (6.3)	Self-reported or previous diagnosis	1) De Jong Gierveld (DG) Loneliness Scale 2) HHIE (social subscale)	Group exercise and socialisation/health education and Group Auditory Rehabilitation	1) The DG emotional loneliness subscale showed greater improvement in the control group: average difference in change of 0.6 (95% CI 0.1 to 1.2; *p* = 0.043; ES = − 0.54). 2) For control and intervention groups, those who attended ≥ 80% of GAR sessions showed significant improvement in DG loneliness scores: Total (95% CI − 2.7 to − 0.9; *p* ≤ 0.001; ES = 1.16) and Emotional (95% CI − 1.7 to − 0.4; *p* = 0.002; ES = 0.96), and HHIE (social: 95% CI − 9.5 to − 0.8; *p* = 0.022; ES = 0.69). The DG social subscale was near significance (95% CI − 1.6 to 0.1; *p* = 0.061; ES = 0.58).
		Follow up (10 weeks)	*n* = 31					
	Control	Baseline	*n* = 31 (17)	M(SD) 74.8 (6.1)			Group Auditory Rehabilitation only	
		Follow up (10 weeks)	*n* = 26					
Nieman et al. 2017 [44]	Intervention	Baseline	*n* = 8	Median (IQR) 69.9 (67.2–79.8)	Clinically significant hearing loss (mild- severe)	UCLA Loneliness Scale- Revised	Hearing screening, device provision and orientation, communication education and counselling	1) Median and mean change in loneliness for both groups was small and varied. 2) Pooled loneliness scores (*n* = 15) indicated a small change from baseline to 3 months post treatment (effect size = 0.31).
		Follow up (1 month, 3 months)	*n* = 8					
	Control	Baseline	*n* = 7	Median (IQR) 72.2 (69.9–74.2)			3-month delay- hearing screening, device provision and orientation, communication education and counselling	
		Follow up (3 months before intervention, 1 month after delated intervention, 3 months after delayed intervention)	*n* = 7

**Table 4:** Repeated measures (pre-post) studies

Study	Baseline sample			Social measure	Intervention	Timepoints	Summary
	Size (Male)	Age M(SD) ***range***	Hearing loss severity				
Applebaum et al. 2019 [ 43]	Cochlear Implant: *N*=51 (-)Hearing Aid: *N*=64 (-)	69 (–) *64.2 −78.5* 71 (–) *63.5-75.8*	Mild to severe	UCLA Loneliness Index-Revised	Cochlear Implant Surgery or Hearing Aids	1)Pre-hearing aid or cochlear implant 2) 6 months post 3) 12 months post 4) 5 years post	Participants who received hearing aids did not show a significant change in loneliness scores over time. For participants who received cochlear implants, loneliness scores significantly reduced at 6-months (mean change: − 3.76, 95% CI − 5.80, − 1.72) and 12-months (mean change: − 2.99, 95% CI − 5.73, − 0.25) from baseline, but were not significantly different from baseline at the 5 year follow-up (mean change: − 0.15, 95% CI − 3.78, 3.48).
Barbosa et al. 2015 [38]	*N* = 125(-)	77.65 (7.6) 65 +	Mild to profound	HHIE - Portuguese*social scale reported separately*	Hearing aid(s) *120 binaural* *5 monaural*	1) Pre-hearing aid fitting 2) at least 4 months after hearing aid fitting (*n* = 125)	A Wilcoxon test indicated that the social scale showed significant improvement post intervention (*P* < 0.001). Pre-hearing aid fitting (M = 20.91, SD = 12.39). Post-hearing aid fitting (M = 8.08, SD = 9.38).
Cuda et al. 2024 [ 46]	*N* = 98 (55)	71.7 (7.6) 60–91	Moderate (*n* = 2) Severe (*n* = 27) Profound (*n* = 69) Mixed (*n* = 7) Sensorineural (*n* = 91)	DG Loneliness Scale	Cochlear implant surgery (Cochlear Ltd.)	1) Baseline (< 2 months pre-surgery) 2) 12 months post-surgery (n = 91) 3) 18 months post-surgery (n = 91)	Odds of higher loneliness scores were significantly lower at 18 months post-surgery compared to baseline (OR 0.38, 95%CI 0.19–0.78). Between baseline and 18 months subjects became significantly less lonely (*p* < 0.01).
Han & Kim, 2024 [36]	*N* = 33 Low Economic Status (LES): 11 (6) Moderate-high economic status (MHES): 22 (9)	LES: 75.45(1.6)– MHES: 74.77(7)–	Moderate to severe (unilateral or bilateral)	LSNS	Hearing aid	1) Baseline – pre hearing aid 2) 6 months post hearing aid (*n* = 33)	For the MHES group, LSNS-18 score (mean difference (MD) = 7.636 10.662, *p* = 0.003), LSNS1 score (MD = 3.136 4.714, *p* = 0.005), and LSNS3 score (MD = 3.045 5.430, *p* = 0.018) showed significant improvement. The LSNS2 score (*p* = 0.138) did not show significant improvement. For the LES group, there was no significant improvement in LSNS-18 scores (*p* = 0.664), LSNS1 score (*p* = 0.092), LSNS2 score (*p* = 0.735), or LSNS3 score (*p* = 0.599).
Lotfi et al., 2009 [ 45]	*N* = 207 (147)	73.01 (8.43) –	Moderate to severe (*n* = 196) Profound (*n* = 9) sensorineural	Hearing Handicap Inventory for the Elderly (HHIE) *social scale reported separately*	Hearing aid	1) Pre-hearing aid fitting 3) 3 months post-hearing aid fitting (n = 207)	Statistically significant improvement in the social subscale from pre-hearing aid fitting (Mean = 35.41, SD = 11.25) to 3 months after hearing aid fitting (Mean = 10.50, SD = 13.69, *p* < 0.000).
Magalhåes et al. 2011 [39]	*N* = 50 (–)	60–74 (n = 24) 75 + (n = 26)	Severe, symmetrical, bilateral, sensorineural	HHIE *social scale reported separately*	Hearing aid fitting, orientation about the use, care and handling of hearing aids, and instruction about communication strategies. Administered over seven meetings	1) Baseline (pre hearing aid) 2) Follow-up (after approx one year of hearing aid use)	For both age groups and genders, there was a significant improvement in social scale scores post intervention in comparison to pre intervention *p* < 0.001. Males showed the greatest amount of improvement.
Uchida et al. 2021 [ 40]	*N* = 94 (39)	76.9 (6.6) 60 +	Mild to moderately severe	1) HHIE *social scale reported separately* 2) Number of Social Activities 3) Convoy Model	Hearing aids	1) Baseline (pre-hearing aid fitting) 2) 6 months post-hearing aid fitting (*n* = 61)	There was a significant improvement in HHIE social scale scores post intervention in comparison to pre intervention, p <.0001. The number of social activities increased slightly from baseline to follow up; however, this increase was not statistically significant, p <.0837. For the convoy model, a significant difference between pre and post intervention was observed only in the total count of whole circles for kin, p <.0344.
Vermeire et al. 2005 [ 37]	Geriatric group: *n* = 25 (–)	74 (–) –	Post-lingually deafened, profound loss	Hearing Handicap Inventory for Adults *social scale reported separately*	Cochlear implant (Cochlear or Med-El)	1) Pre-surgery 2) Follow-up (n = 10; mean follow-up period is 16 months)	The social scale showed significant improvement post intervention (t = 4.958, *p* = 0.001). Pre-surgery (M = 79, SD = 21. 89). Follow-up (M = 57, SD = 24.53).
Weinstein et al. 2016 [ 42]	*N* = 40 (14)	80.4 (7.2) 62–92	Mild to moderately severe	DG Loneliness Scale	Hearing aids (Widex 30%, Oticon, 37%, Unitron 10%, Siemens 5%, Starkey 5%, Resound 3%, Phonak 3%)	1) Baseline (Pre-hearing aid fitting) 2) Post hearing aid fitting (4–6 weeks after hearing aid fitting; *n* = 40)	Significantly lower scores were observed at follow-up than baseline for overall loneliness (*p* <.001, effect size: *r* = -.39) and emotional loneliness (*p* <.05, effect size: *r* = -.28). Social loneliness scores also reduced (but were not statistically significant). When results compared between hearing loss groups (mild vs. moderate-severe), only the moderate-severe group showed significant improvement in total loneliness (*p* <.01, effect size: *r* = −.414) and emotional loneliness (*p* <.05, effect size: *r* = −.311).

The included studies were conducted in a variety of locations worldwide including Europe [36, 37], Brazil [38, 39], Japan [40], North America [41–44],and Iran [45]. One study was multinational, including patients from Cochlear Implant Centres across Italy, Spain, Australia, and Israel [46]. Two RCTs were included [41, 44]. The remaining 9 studies were prospective, pre-test-post-test studies. Baseline sample sizes ranged from 10 [37] to 207 [45].

Two studies had participants with one consistent level of hearing loss: severe (39) and profound (37). Other studies reported participant samples with a range of hearing loss severity levels: mild to moderate [40, 42], mild to severe [43, 44], mild to profound [38], moderate to profound [46], and moderate to severe [36, 45]. One study did not specify hearing loss severity, although participants were identified as diagnosed with hearing loss [41]. The majority of interventions focused on hearing device uptake, including hearing aids, cochlear implants, and assistive listening technology. The effectiveness of Group Auditory Rehabilitation (GAR) was also evaluated. The majority of studies targeted loneliness or social participation as outcome variables, with only 1 study targeting social isolation as an outcome variable.

### Loneliness

Four studies measured the impact of interventions on loneliness in older adults with hearing loss [41–43, 46], using the De Jong Gierveld (DG) Loneliness scale as a loneliness outcome measure [47]. The DG Loneliness Scale includes two subscales: emotional loneliness (e.g., felt deficit in intimate attachment; 5 items) and social loneliness (e.g., absence of a broader engaging social network; 6 items). Total scale scores range from 0 (not lonely) to 11 (extremely lonely). Two studies [43, 44] used the 20 item UCLA Loneliness Index Revised [48, 49]. This measure asks participants to indicate how often they feel ‘left out’ or ‘isolated from others,’ with response options of ‘never,’ ‘rarely,’ ‘sometimes,’ or ‘often.’ The index is scored from 0–80 with lower scores indicating less loneliness [48, 49].

Two pilot RCTs included loneliness as an outcome measure [41, 44]. In Jones et al. [41], the intervention group completed an exercise and socialization/health education course combined with GAR (*n* = 31). The control group (*n* = 26) completed only the GAR. The results indicated that the emotional loneliness score on the DG loneliness scale showed greater improvement for the control group, with an average difference in change of 0.6 (95% CI 0.1 to 1.2; *p* = 0.043; ES = − 0.54). For both control and intervention groups, participants who attended ≥ 80% of GAR sessions showed improvement in DG loneliness scores, Total (95% CI − 2.7 to − 0.9; *p* ≤ 0.001; ES = 1.16) and Emotional (95% CI − 1.7 to − 0.4; *p* = 0.002; ES = 0.96). A tendency was also observed for the social subscale, although not statistically significant (95% CI − 1.6 to 0.1; *p* = 0.061; ES = 0.58).

The comprehensive GAR sessions were delivered over 10 weeks (3 h per week) and included sessions on communication goal setting, communication strategies, coping with hearing loss, handling difficult listening situations, types and uses of hearing assistive technologies, local resources, advocating for yourself and others with hearing loss, hands on support with existing hearing technology (*e.g.*, hearing aids), and mindfulness and stress-reduction strategies. Participants engaged in practical exercises as a group and at home with communication partners (*e.g.*, spouse, significant other or friend). In addition to these sessions, a single 3-h session was scheduled specifically with communication partners. While audiologists lead the hearing technology sessions, most of the GAR was facilitated by trained students and the principal research investigator.

Additionally, Nieman et al. [44] piloted an RCT on the effectiveness of an over-the-counter assistive listening device intervention. The intervention was a one-time session with a trained interventionist, the person with hearing loss, and a communication partner (e.g., family member). The session involved selecting an assistive listening device (i.e., a personal amplification system), fitting and orientation to the device, education on hearing loss, and “aural rehabilitation with a focus on communication strategies and expectation management” [44]. Participants were assigned to an immediate intervention group or a 3 month delayed intervention group, with UCLA Loneliness Index Revised scores measured before and after intervention. Sample sizes for this study were small (*n* = 8 for immediate intervention; *n* = 7 for delayed intervention). The results showed small and varied reductions in loneliness scores for both intervention groups three months post treatment in comparison to baseline.

The remaining studies measuring loneliness applied a pre-post design. One of these studies evaluated loneliness before and after cochlear implant surgery [46]. Cuda et al. [46] showed a statistically significant reduction in loneliness between baseline (less than two months before surgery) and 18 months post-surgery (*p* < 0.01, *n* = 91). An additional study evaluated a hearing aid intervention [42]. Weinstein et al. [42] showed statistically significant reductions in overall loneliness (*p* < 0.001, effect size: r = −0.39) and emotional loneliness (*p* < 0.05, effect size: r = −0.28) 4–6 weeks after hearing aid fitting (*N* = 40). Social loneliness scores also trended in this direction but were not statistically significant.

One additional study included participants who qualified for both hearing aids and Cochlear Implants in their longitudinal study on loneliness [43]. The results from Applebaum et al. [43] indicated that participants who received hearing aids (*n* = 64) did not show a significant change in UCLA loneliness scores over time (6 months, 12 months, and 5-year follow-up). Cochlear Implant recipients’ (*n* = 51) loneliness scores reduced at 6 and 12 months in comparison to baseline scores (6 months mean change: − 3.76, 95% CI − 5.80, − 1.72; 12 months mean change: − 2.99, 95% CI − 5.73, − 0.25), but were similar to baseline at a 5-year follow-up assessment (mean change: − 0.15, 95% CI − 3.78, 3.48).

### Social isolation

In comparison to research with loneliness or social participation as outcome variables, social isolation had the least amount of research available, with only one study that included statistical analysis [36]. Han and Kim [36] used the Lubben Social Network Scale (LSNS) to evaluate effects of a hearing aid intervention on social isolation in older adults with hearing loss. The LSNS assesses perceived support received from family and friends [50]. Items measure size (named as LSNS1), closeness (LSNS2) and frequency of contact (LSNS3) of a respondent’s family, friends, and neighborhood. The total score ranges from 0 to 50, with a lower score indicating a greater risk of isolation. Han and Kim [36] tested the effect of a hearing aid fitting intervention on LSNS for older adults with a low or moderate to high socioeconomic status (SES). The results indicated that the total score, LSNS1 and LSNS3 showed improvement in the moderate-high SES group (total: *p* = 0.003 LSNS1: *p* = 0.005, LSNS3: *p* = 0.018) but not in the low SES group.

### Social participation

Six studies evaluated social participation in older adults with hearing loss before and after interventions [37–41, 45] using the social subscale of the Hearing Handicap Inventory for the Elderly (HHIE). Scores from this 12-item subscale range from 0–48, with higher scores indicating greater restriction in social participation due to hearing loss. In their RCT, Jones et al. [41] reported improvements in the HHIE social subscale for both participants in the intervention group (exercise and health education with Group Auditory Rehabilitation) and control group (Group Auditory Rehabilitation alone) who attended ≥ 80% of auditory rehabilitation sessions (*p* = 0.022). Group Auditory Rehabilitation (GAR) is a structured, group-based intervention that combines education, communication skills training, and hearing-technology support to improve communication and coping among adults with hearing loss. Among the four studies that used the HHIE to assess the effectiveness of a hearing aid fitting intervention [38–40, 45], all showed improvements in social participation over time, as measured by the social subscale of the HHIE (*p* < 0.001 for all studies). Vermeire et al. [37] also used the HHIE to evaluate the effectiveness of a cochlear implant intervention and showed improvement in social participation 16 months post-surgery (*p* = 0.001).

### Quality assessment

Using the Cochrane Risk of Bias tool, Jones et al. [41] and Nieman et al. [44] showed low risk of bias in all five domains (randomization, deviations from intended interventions, missing outcome data, measurement of outcome, and selection of reported results). The final quality assessment rating for all non-RCT studies was serious risk of bias due to risk of confounding comorbidities. Cognitive decline, depression, and vision loss, were identified as potential confounding factors due to associations between these variables and higher levels of social isolation, loneliness and restrictions in social participation [51]. Although some studies measured relevant comorbidities as part of exclusion criteria or as additional pre-post measures, none controlled for or evaluated how comorbidities affected change in loneliness, social isolation, or social participation over time. One study included in the review measured symptoms of depression [36]. Cognitive skills were measured as outcome variables before and after intervention for 3 studies [36, 39, 40] showing stability or improvement in cognitive functioning with hearing aid intervention. Cognitive decline was stated as an exclusion criteria for 3 studies [37, 40, 42]. Two studies did not consider comorbidities as part of the study design [38, 45] and none measured visual acuity.

## Discussion

This review aimed to explore the effectiveness of interventions for social isolation, loneliness, and social participation in older adults with hearing loss, and evaluate if these include intervention programs other than hearing technology uptake alone. We found that for research focusing on older adults with hearing loss, interventions for social isolation, loneliness, and social participation almost exclusively focused on correcting the hearing loss with hearing aids or cochlear implants, or adjustments to these assistive technologies during uptake, with one intervention design evaluating GAR combined with a exercise and socialization/health education course [41], and one other intervention incorporating over-the-counter assistive listening devices [44].

The majority of studies included in the review showed improvements in social isolation, loneliness, and social participation post-intervention. In the present review, two studies employed a randomized controlled trial design, both of which were pilot studies with limited sample sizes. Although often considered the strongest design for intervention research, RCTs with longitudinal measurement can be costly if long term follow ups are monitored and may warrant additional consideration when not pragmatic and applied to outcomes such as loneliness, social participation, and social isolation. As highlighted by Levasseur et al. [52] pragmatic RCTs conducted in community contexts must balance methodological rigor with feasibility, acceptability, and implementation constraints, particularly when interventions are delivered through community organizations and rely on sustained participant engagement over time. Consequently, longitudinal observational and pretest–posttest designs provide valuable complementary evidence regarding intervention effectiveness under real-world conditions. Findings of this review therefore support that a decline in social isolation and loneliness, and increases in social participation were observed at follow-up periods extending beyond six months in several pretest–posttest design studies [37, 39, 46]. For example, Cuda et al. [46] reported reductions in loneliness that persisted for at least 18 months following cochlear implant surgery, suggesting that intervention-related improvements may be sustained over time.

The pilot RCT conducted by Jones et al. [41] demonstrated feasibility and acceptability of a GAR intervention for older adults with hearing loss, and provides preliminary evidence that collaborative, community-based GAR sessions that include communication partners can help to address loneliness and restriction in social participation of older adults with hearing loss. Although this study did not find an additional benefit of group exercise on loneliness or social participation, there was a benefit found for physical fitness outcomes (e.g., balance). Overall, the results of this study suggest that consistent GAR attendance alone can be an effective intervention protocol to address loneliness and restriction in social participation.

Han and Kim [36] made an additional important contribution to the literature by conducting formal analysis on a measure of social isolation (Lubben Social Network Scale) before and 6 months after a hearing aid intervention. A unique aspect of this study is the interaction between change in LSNS scores over time and the socioeconomic status (SES) of older adults with hearing loss. That is, the Moderate-High SES group showed reductions in social isolation 6 months post hearing aid fitting, whereas the low SES did not show improvement in social isolation. This interaction between SES, social isolation, and hearing aid uptake should continue to be investigated in future research. Indeed, although additional research is required for all outcome variables, this review showed that intervention studies including standardized measures of social isolation had the least amount of research available at this time. This suggests that social isolation is a particularly important area for future research.

### Future directions

To capture both short-term efficacy and longer-term effectiveness of diverse interventions targeting loneliness, social isolation, and social participation, future research should use a range of complementary designs, including RCTs with larger sample sizes, realist evaluation and longitudinal observational studies. When using RCTs, pragmatic designs, longer follow-up periods, retention strategies, and measurement of contextual factors (e.g., comorbidities, social environment) and implementation outcomes are particularly important for addressing sources of bias identified in the current literature. Additional research that explicitly and consistently incorporates validated measures of social isolation is warranted, given its comparatively limited representation in the existing intervention literature.

Future technology focused intervention studies should follow up Nieman et al. [44] by evaluating the effects of assistive listening devices (e.g., pocket talkers) on social isolation, loneliness, and social participation in older adults with hearing loss, as these devices are significantly less expensive and invasive than hearing aids and cochlear implant surgery, and may therefore be more accessible to the general older adult population. As cognitive decline, vision loss, and depression are prevalent among older adults, and are also strongly associated with social isolation, loneliness and social participation, future research should consistently integrate co-morbidities into study designs and statistical analyses. The potential additive effects of vision loss, hearing loss and cognitive decline on social isolation, loneliness, and social participation, and the effects of intervention on older adults with dual-sensory impairment (i.e., hearing and vision loss), are important avenues for future research. Additionally, although the HHIE is a standardized and validated measure often used in hearing loss research, including additional social participation measures, such as the Elderly Activity Inventory Questionnaire [53] could benefit the generalizability of results.

Since completion of this review, findings from secondary analyses of the Aging and Cognitive Health Evaluation in Elders (ACHIEVE) trial, a large, multicentre randomized clinical trial of 977 older adults with untreated hearing loss, have been published and provide important context for the current study [54]. Compared with a health education control group, participants in a best-practice hearing intervention (four audiologist-led sessions over 8 weeks, hearing aids, counseling, and education, with booster sessions every six months) demonstrated reduced social isolation over three years, reflected by greater retention of social network size, diversity, and embeddedness. The intervention was also associated with a modest attenuation of increases in loneliness over time. Together, these findings suggest that hearing interventions may help preserve social connections and slow the worsening of loneliness in older adults. The results also highlight that reductions in loneliness may require intervening over a longer duration and on additional psychosocial components beyond hearing.

## Limitations

Although this review is unique in that it includes different types of interventions, such as hearing aids, cochlear implants, GAR, and over-the-counter hearings devices, this review does have a few limitations. Due to the limited amount of intervention research including older adults with hearing loss, we included non-randomized studies. While previous studies were mainly non-randomized pretest–posttest designs, these approaches contribute to examining longitudinal change in real-world clinical and community settings. However, the absence of control groups and inconsistent adjustment for comorbidities limits generalization of the results due to threats to internal validity, and additional contextual factors of significance. Moreover, differences in the severity of hearing loss of participants, the variety of outcome measures, and the lack of consistent evaluation of co-morbidities made interpretation and comparison between studies difficult. Additionally, although the review included studies from many different countries, the exclusion of non-English studies may also be a source of bias for this review.

## Conclusions

The best available evidence indicates that hearing aid and cochlear implant intervention generally show improvement in loneliness and social participation in older adults with hearing loss, with less research available specifically for social isolation. Although this evidence is predominantly derived from non-randomized pretest–posttest design studies, an initial pilot RCT also supported the effectiveness of Group Auditory Rehabilitation (GAR) in reducing loneliness and restrictions in social participation. This RCT suggests that goal setting, communication and stress-reduction strategies to adapt to hearing loss, alongside consistent use of hearing assistive technologies, may hold promise for addressing social isolation, loneliness, and restrictions in social participation. Further research would help to support existing evidence by incorporating pragmatic designs, longer follow-up periods, retention strategies and measurement of contextual factors (e.g., comorbidities and social environment).

## Data Availability

Data sharing not applicable to this article as no datasets were generated or analysed during the current study.

## Appendix

### Embase

**Table:**

#	Query	Results (Jan 25, 2024)
1	(seniors or elderly or older persons or older adults or gerontolog* or geriatr* or aging).ti,ab.	850,968
2	exp aged/or exp aging/or exp gerontology/or exp geriatrics/	3,970,532
3	1 or 2	4,178,076
4	(deaf* or (hearing adj3 (impair* or loss or disorder))).ti,ab.	117,600
5	exp hearing impaired person/or exp hearing impairment/or exp hearing disorder/	162,733
6	4 or 5	199,186
7	(intervention or program evaluation or trial or hearing rehabilitation or hearing aid or cochlear implant or assistive listen*).ti,ab.	2,182,818
8	exp intervention study/or exp program evaluation/or exp clinical trial/or exp auditory rehabilitation/or exp hearing aid/or exp assistive listening device/or exp cochlea prosthesis/	1,991,242
9	7 or 8	3,403,104
10	(loneliness or social isolation or social participation or social engagement or social activit* or social network* or social interaction* or social disengagement or social alienation).ti,ab.	105,106
11	exp loneliness/or exp social isolation/or exp social interaction/or exp social participation/	126,208
12	10 or 11	185,211
13	3 and 6 and 9 and 12	345
14	limit 13 to (English language and yr="2000 -Current")	312

### Medline

**Table:**

#	Query	Results (Jan 25, 2024)
1	(seniors or elderly or older persons or older adults or gerontolog* or geriatr* or aging).ti,ab.	638,837
2	exp aged/or exp aging/or exp geriatrics/	3,690,689
3	1 or 2	3,892,759
4	(deaf* or (hearing adj3 (impair* or loss or disorder))).ti,ab.	100,218
5	exp hearing loss/or exp hearing disorders/or exp deafness/	98,262
6	4 or 5	137,766
7	(intervention or program evaluation or trial or hearing rehabilitation or hearing aid or cochlear implant or assistive listen*).ti,ab.	1,484,795
8	exp program evaluation/or exp clinical trial/or exp hearing aids/or exp cochlear implants/	1,086,931
9	7 or 8	2,143,049
10	(loneliness or social isolation or social participation or social engagement or social activit* or social network* or social interaction* or social disengagement or social alienation).ti,ab.	86,269
11	exp loneliness/or exp social isolation/or exp social participation/or exp social interaction/	31,137
12	10 or 11	104,925
13	3 and 6 and 9 and 12	191
14	limit 13 to (English language and yr="2000 -Current")	162

### CINAHL

**Table:**

#	Query	Results (Jan 25, 2024)
S1	seniors OR elderly OR "older persons" OR "older adults" OR gerontolog* OR geriatr* OR aging	263,272
S2	"hearing loss" OR deaf* OR "hearing impair*" OR "hard of hearing" OR "hearing disorders"	51,494
S3	intervention OR "program evaluation" OR trial OR "hearing rehabilitation" OR "hearing aid" OR "cochlear implant" OR "assistive listen*"	945,699
S4	loneliness OR "social isolation" OR "social participation" OR "social engagement" OR "social activit*" OR "social network*" OR "social interaction*" OR "social disengagement" OR "social alienation"	79,862
S5	S1 AND S2 AND S3 AND S4	135

Limiters on all searches include: Publication Date: 20000101- 20241231; English Language; Peer Reviewed

### Ageline

**Table:**

#	Query	Results (Jan 25, 2024)
S1	"hearing loss" OR deaf* OR "hearing impair*" OR "hard of hearing" OR "hearing disorders"	1,080
S2	intervention OR "program evaluation" OR trial OR "hearing rehabilitation" OR "hearing aid" OR "cochlear implant" OR "assistive listen*"	31,164
S3	loneliness OR "social isolation" OR "social participation" OR "social engagement" OR "social activit*" OR "social network*" OR "social interaction*" OR "social disengagement" OR "social alienation"	7,693
S4	S1 AND S2 AND S3	35

Limit on all searches: Publication Date: 20000101- 20241231; Peer Reviewed; English language

### PsycINFOInfo

**Table:**

#	Query	Results (Jan 25, 2024)
S1	seniors OR elderly OR "older persons" OR "older adults" OR gerontolog* OR geriatr* OR aging	196,995
S2	"hearing loss" OR deaf* OR "hearing impair*" OR "hard of hearing" OR "hearing disorders"	17,758
S3	intervention OR "program evaluation" OR trial OR "hearing rehabilitation" OR "hearing aid" OR "cochlear implant" OR "assistive listen*"	490,624
S4	loneliness OR "social isolation" OR "social participation" OR "social engagement" OR "social activit*" OR "social network*" OR "social interaction*" OR "social disengagement" OR "social alienation"	111,177
S5	S1 AND S2 AND S3 AND S4	77

Limit on all searches: Publication Year: 2000-2024; Peer Reviewed; English language

### ProQuest Sociology

**Table:**

#	Query	Results (Jan 30, 2024)
S1	noft(seniors OR elderly OR "older persons" OR "older adults" OR gerontolog* OR geriatr* OR aging) AND PEER(yes) AND pd(>20001231)	135581
S2	noft("hearing loss" OR deaf* OR "hearing impair*" OR "hard of hearing" OR "hearing disorders") AND PEER(yes) AND pd(>20001231)	3609
S3	noft(intervention OR "program evaluation" OR trial OR "hearing rehabilitation" OR "hearing aid" OR "cochlear implant" OR "assistive listen*") AND PEER(yes) AND pd(>20001231)	264805
S4	noft(loneliness OR "social isolation" OR "social participation" OR "social engagement" OR "social activit*" OR "social network*" OR "social interaction*" OR "social disengagement" OR "social alienation") AND PEER(yes) AND pd(>20001231)	77596
S5	S4 AND S3 AND S2 AND S1	17
